# Mitochondria-Dependent Metabolic Reprogramming Enhances Myofibroblast Differentiation and Aggravates Bleomycin-Induced Pulmonary Fibrosis

**DOI:** 10.3390/cells15070582

**Published:** 2026-03-25

**Authors:** Kai Yazaki, Yosuke Matsuno, Yuki Yabuuchi, Sosuke Matsumura, Kenya Kuramoto, Kazufumi Yoshida, Masashi Matsuyama, Takumi Kiwamoto, Yuko Morishima, Yukio Ishii, Kaori Ishikawa, Kazuto Nakada, Nobuyuki Hizawa

**Affiliations:** 1 Department of Respiratory Medicine, Institute of Medicine, University of Tsukuba, Tsukuba 305-8575, Japan; 2 Department of Respiratory Medicine, Nikko Memorial Hospital, Hitachi 317-0055, Japan; 3 Department of Respiratory Medicine, National Hospital Organization Ibarakihigashi National Hospital, Tokai 319-1113, Japan; 4 Institute of Life and Environmental Sciences, University of Tsukuba, Tsukuba 305-8572, Japan

**Keywords:** mitochondrial dysfunction, pulmonary fibrosis, fibroblast, macrophage

## Abstract

Idiopathic pulmonary fibrosis (IPF) is a progressive interstitial lung disease characterized by irreversible fibrosis. Aberrant cell differentiation plays a crucial role in the development of IPF. Although recent studies have suggested that mitochondrial dysfunction may play a role in IPF, its direct impact on fibrosis remains unclear. This study aimed to clarify the role of mitochondria in lung cell differentiation and pulmonary fibrosis development by employing mito-mice ND6^M^, in which the activity of respiratory chain complex I is decreased due to a mitochondrial DNA mutation (G13997A). Pulmonary fibrosis was induced by administering bleomycin (BLM) to both wild-type and mito-mice ND6^M^. Bone marrow-derived macrophages and primary lung fibroblasts, generated from both types of mice, were analyzed to evaluate M1/M2 polarization and myofibroblast differentiation, respectively. Compared to wild-type mice, mito-mice ND6^M^ exhibited more severe fibrosis and lower survival rates following BLM inoculation. Lactate production in the lungs after BLM administration was significantly higher in mito-mice ND6^M^ than in wild-type mice. TGF-β1-treated fibroblasts from mito-mice ND6^M^ exhibited increased α-smooth muscle actin expression. While type I collagen expression was not different between these mice, TGF-β1-induced expression of phosphoserine phosphatase and serine hydroxymethyltransferase2, two of the enzymes involved in the serine–glycine pathway, was significantly higher in mito-mice ND6^M^ than in wild-type mice. On the other hand, mitochondrial dysfunction had a small effect on pulmonary inflammation and on M1/M2 macrophage polarization. In conclusion, mitochondrial dysfunction promotes TGF-β1-induced myofibroblast differentiation and BLM-induced pulmonary fibrosis. Mitochondria-dependent metabolic reprogramming may therefore represent a promising therapeutic target in IPF.

## 1. Introduction

Idiopathic pulmonary fibrosis (IPF) is a lung disease with a poor prognosis [1]. The pathogenesis of IPF is believed to result from chronic injury of the alveolar epithelium with subsequent excessive repair and progressive fibrosis [2]. The development of IPF is associated with abnormal cell differentiation, such as differentiation of fibroblasts into myofibroblasts and M2 polarization of alveolar macrophages [2,3]. Elucidating the mechanisms of pathogenetic cell differentiation is important to control the progression of IPF.

The American Thoracic Society (ATS)/European Respiratory Society (ERS)/Japanese Respiratory Society (JRS)/Latin American Thoracic Association (ALAT) guidelines state that IPF occurs mainly in the elderly over 60 years of age, suggesting the association of senescence with its pathogenesis [1]. Senescence-related phenotypic changes, including telomere shortening, genomic instability, autophagy, and mitochondrial dysfunction, are demonstrated in IPF [4,5]. However, it is not yet clear whether these changes are a cause or a consequence of IPF.

Previous reports have shown the involvement of aberrant lung cell differentiation in the pathogenesis of IPF. Macrophages polarize into M1 and M2. M1 macrophages produce proinflammatory cytokines and act in defense against infection. M2 macrophages have roles in anti-inflammation and wound healing, and contribute to the development of IPF [3]. Fibroblasts play a central role in the progression of IPF through their differentiation into myofibroblasts characterized by α-smooth muscle actin (α-SMA) expression and overproduction of the extracellular matrix (ECM) [6]. Reports have shown that cell differentiation is dependent on cellular metabolism. In the lungs of IPF, decreased oxidative phosphorylation (OXPHOS), the process of producing ATP through the respiratory chain complexes, increases the production of lactate, which induces the differentiation of fibroblasts into myofibroblasts [7,8]. OXPHOS is necessary for the polarization of M2 macrophages [9]. Furthermore, reactive oxygen species (ROS) play a crucial role in TGF-β1-induced myofibroblast differentiation [10,11] and epithelial–mesenchymal transition [12]. Mitochondria not only play a role in intracellular energy production and homeostasis, but also regulate the production of various metabolites [13]. While mitochondria may modulate cell differentiation by regulating cellular metabolism, their role in this process remains elusive.

Analyzing the role of mitochondria was difficult because methods for genetic manipulation of mitochondrial DNA (mtDNA) were not established. In recent years, mice carrying mutant mtDNA have been developed, enabling the functional analysis of mitochondria [14]. mtDNA is classified into genes for proteins, ribosomal RNA, and transfer RNA, which are mainly involved in the electron transfer system [15]. The electron transfer system consists of respiratory chain complexes I-V and synthesizes ATP. Electrons leaked from this system react with oxygen to generate ROS. Mito-mice ND6^M^ have mtDNA mutation G13997A in the *ND6* gene, which encodes one of the subunits of the respiratory chain complex I [16,17]. The mice possessing only G13997A were generated by performing the intercellular transfer of G13997A mtDNA from highly metastatic tumor cells into mouse embryonic stem cells. Sharing the nuclear background of C57L/6J mice excludes the involvement of nuclear DNA mutations developed in mito-mice ND6^M^. The mice show decreased activity of respiratory complex I and the overproduction of lactate and ROS [16,18].

Metabolic reprogramming due to mitochondrial dysfunction may exacerbate pulmonary fibrosis via abnormal lung cell differentiation. The aim of this study is to elucidate the role that mitochondria-dependent metabolic reprogramming plays in lung cell differentiation and the development of pulmonary fibrosis through the analysis of mito-mice ND6^M^.

## 2. Methods

### 2.1. Animals

All of the C57BL/6J mice (wild-type mice) and mito-mice ND6^M^ used in this study were 8-week-old females. Wild-type mice were purchased from Charles River Laboratories, Japan. Mito-mice ND6^M^ were provided by Dr. Kaori Ishikawa and Dr. Kazuto Nakada of the Institute of Life and Environmental Sciences, University of Tsukuba. All animal experiments were approved by the University of Tsukuba Institutional Animal Care and Use Committee (Approval Code: 24-312. Approval Date: 10 June 2024).

### 2.2. Bleomycin (BLM)-Induced Pulmonary Fibrosis

Mice were administered 0.1 mg/kg BLM (Cayman, Ann Arbor, MI) or normal saline (NS) by oropharyngeal aspiration. The administration was performed once daily for 6 consecutive days [19]. BLM-induced lung injury is a biphasic model consisting of an initial inflammatory phase followed by a fibrotic phase. Pulmonary inflammation predominates during the first week after bleomycin administration, whereas lung fibrosis develops and becomes prominent by day 14 [20,21]. Mice were sacrificed on day 8 and day 14 for the analysis of BLM-induced pulmonary inflammation and fibrosis, respectively.

### 2.3. Pathology

Paraffin sections of lungs were stained with hematoxylin–eosin (40× and 100× magnification) and Masson’s trichrome (100× magnification). The severity of pulmonary fibrosis was quantified using the Ashcroft score [22]. A total of 30 randomly selected fields from lung sections were observed at 100× magnification and scored with a grade from 0 to 8. The mean of all fields was evaluated as the pathological score.

### 2.4. Analysis of Collagen Synthesis

Hydroxyproline is a major component of collagen and is used as an indicator of collagen synthesis. Lungs were removed and homogenized on day 14 of BLM administration. Hydroxyproline content was determined using the Hydroxyproline Colorimetric Assay Kit (BioVision, Milpitas, CA, USA) according to the manufacturer’s protocol.

### 2.5. Bronchoalveolar Lavage (BAL)

BAL was performed on day 8 of BLM administration. Lungs were lavaged with 5 sequential 1 mL aliquots of NS. The supernatant of the first BAL fluid was used to measure protein levels. Total cells, including the first BAL fluid pellet, were counted using a hemocytometer. Differential cell counts were performed by staining Diff-Quick (Sysmex, Kobe, Japan).

### 2.6. Lactate Assay

Lung tissue (10 mg) was homogenized in 100 μL of lactate assay buffer supplied with the Lactate Colorimetric Assay Kit II (BioVision). To obtain the supernatant for the assay, the lysate was centrifuged at 10,000× *g* for 10 min. The supernatant was treated according to the manufacturer’s protocol, and then the lactate levels were determined using Varioskan Lux (Thermo Fisher Scientific, Waltham, MA, USA).

### 2.7. Bone Marrow-Derived Macrophages (BMDMs)

Bone marrow cells were obtained from the femur and tibia of mice. Cells were differentiated into macrophages by incubation with Dulbecco’s modified Eagle’s medium (DMEM) + 20% fetal bovine serum (FBS) + 1% penicillin/streptomycin + 30% culture supernatant of L929 cells producing M-CSF. BMDMs were cultured overnight in 12-well plates at a density of 4 × 10^5^ cells in 1 mL/well of medium. Cells were treated with 50 ng/mL of IFN-γ (Peprotech, Cranbury, NJ, USA) and 100 ng/mL of lipopolysaccharide (LPS) (Santa Cruz Biotechnology, Santa Cruz, CA, USA), 20 ng/mL of IL-4 (Peprotech), or culture medium alone for 24 h.

### 2.8. Primary Lung Fibroblasts

Mouse lung tissue was minced and digested with 75 U/mL collagenase (Wako, Osaka, Japan) at 37 °C for 30 min. Primary fibroblasts were obtained by culturing isolated lung cells in medium for 7 days after 3 washes in ice-cold phosphate-buffered saline. To induce myofibroblast differentiation, cells were cultured in 12-well plates at a density of 2 × 10^4^ cells in 1 mL/well of medium for 4 days. The cells were then incubated in medium with 0.5% FBS for 24 h and then treated with 5 ng/mL of recombinant mouse TGF-β1 (R&D Systems, Minneapolis, MN, USA) for 72 h.

### 2.9. RNA Extraction and Real-Time Reverse Transcription PCR (Real-Time RT-PCR)

Total RNA was extracted from lung tissue using the RNeasy kit (QIAGEN, Hilden, Germany) and from BMDMs and primary fibroblasts using TRIzol (Invitrogen, Carlsbad, CA, USA). Total RNA (1 µg) was converted to cDNA. Real-time RT-PCR was performed using the QuantStudio 5 Real-Time PCR System (Thermo Fisher Scientific). Primer sequences used in real-time RT-PCR are presented in Table 1. Results were analyzed using the ΔΔCT method and expressed as a ratio to the expression level of GAPDH.

### 2.10. Western Blot Analysis

Proteins were extracted by lysing lung tissue in RIPA Lysis Buffer (Santa Cruz Biotechnology, Santa Cruz, CA, USA) containing PMSF and a protease inhibitor. The protein concentrations were measured using the Bradford assay. The lysates were mixed with SDS sample buffer and boiled at 95 °C for 5 min. The proteins were separated by 5–20% SDS–PAGE in 25 mM Tris, 192 mM glycine, 0.1% SDS buffer. The proteins were electrically transferred onto PVDF membranes in 25 mM Tris, 192 mM glycine, and 20% methanol buffer. Following the manufacturer’s instructions, blots were blocked with Odyssey blocking buffer (LI-COR Biosciences, Lincoln, NE, USA). The blots were incubated overnight at 4 °C with the primary antibodies against α-SMA (1:1000; Abcam, Cambridge, UK), type I collagen (1:1000; Abcam), fibronectin (1:500; Abcam), and β-tubulin (1:500; Abcam). After incubation with the secondary antibody (IRDye^®^ 680RD donkey anti-rabbit IgG, 1:10,000; LI-COR Biosciences), the proteins were visualized using the Odyssey Imaging System (LI-COR Biosciences). The band intensities were normalized to β-tubulin.

### 2.11. Statistical Analysis

Results were expressed as means (±SD). After confirming normality using the Shapiro–Wilk test, data comparisons among groups were performed by one-way ANOVA followed by Tukey’s test. Survival analysis was performed using the Kaplan–Meier method and the log-rank test. Graphpad Prism 8 (GraphPad Software, La Jolla, CA, USA) was used for statistical analysis. *p*-values < 0.05 were considered statistically significant.

## 3. Results

### 3.1. Mitochondrial Dysfunction Decreases Survival in BLM-Induced Pulmonary Fibrosis Model

**Figure 1:**
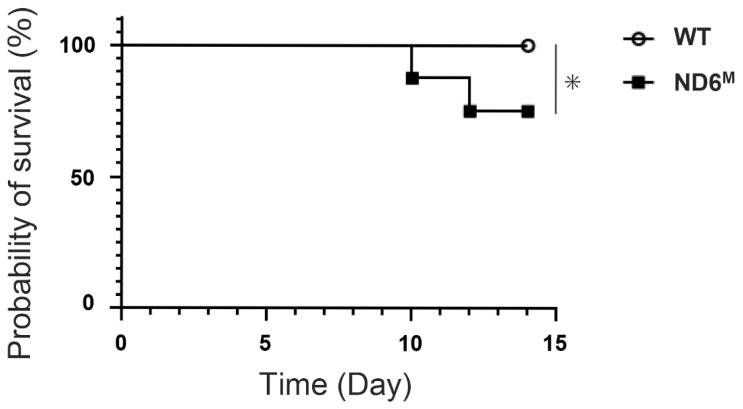
Survival following bleomycin (BLM) inoculation is decreased in mito-mice ND6^M^. Kaplan–Meier survival curves for wild-type (WT) and mito-mice ND6^M^ (ND6^M^) after administration of 0.1 mg/kg BLM. The data represent the probability of survival in each group (*n* = 16). * *p* < 0.05.

To investigate the effect of mitochondrial dysfunction on the severity of BLM-induced pulmonary lesions, we compared the survival between wild-type and mito-mice ND6^M^. Only mito-mice ND6^M^ died on day 10 or later after BLM treatment and had a significantly lower survival rate compared to wild-type mice (Figure 1). This result indicates that mitochondrial dysfunction significantly aggravates lung pathology and affects survival in the BLM-induced lung fibrosis model.

### 3.2. Mitochondrial Dysfunction Enhances BLM-Induced Pulmonary Fibrosis

**Figure 2:**
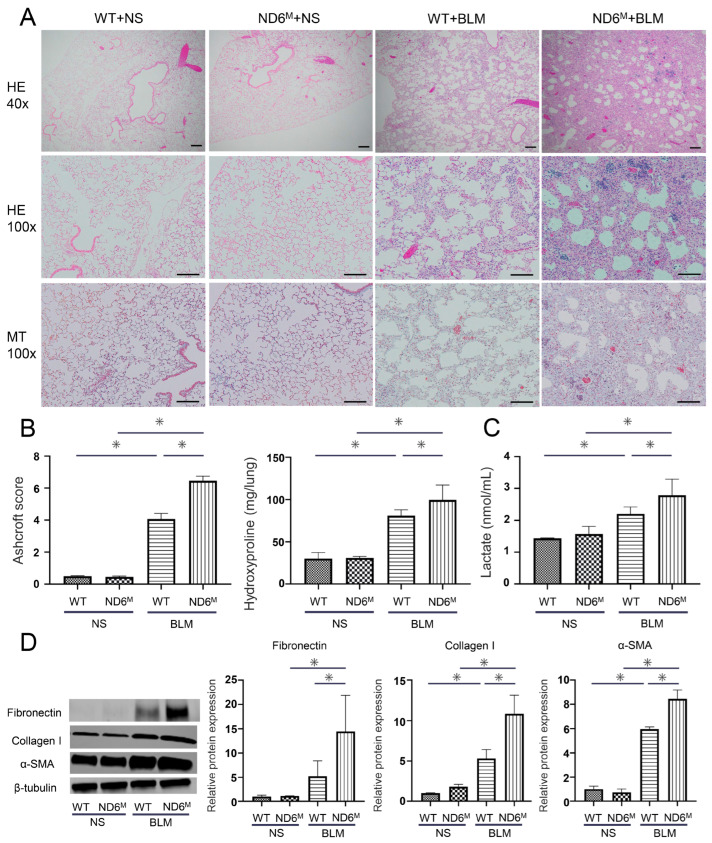
Bleomycin (BLM)-induced pulmonary fibrosis and lactate production are enhanced in mito-mice ND6^M^. Wild-type (WT) and mito-mice ND6^M^ (ND6^M^) were administered 0.1 mg/kg BLM or normal saline (NS) via oropharyngeal aspiration. The mice were then analyzed 14 days later. (**A**) Representative photomicrographs of the lungs stained with hematoxylin and eosin (magnification ×40 and ×100) and Masson’s trichrome (magnification ×100). Scale bars = 200 µm (×40) and 100 µm (×100). (**B**) The Ashcroft score based on pathological findings and hydroxyproline content in the lungs. (**C**) The lactate levels in the lungs. (**D**) The protein expression of fibronectin, type I collagen, and α-smooth muscle actin (α-SMA) in the lungs. β-tubulin was used as a loading control. Data are representative of two independent experiments and shown as the mean ± SD (*n* = 8). * *p* < 0.05.

Next, we examined the effect of mitochondrial dysfunction on the development of pulmonary fibrosis following BLM administration. On histological examination of wild-type mice, BLM caused the thickening of the alveolar septa with fibrosis (Figure 2A). The alveolar structure was relatively preserved. In mito-mice ND6^M^, BLM caused more severe fibrosis with the distortion of alveolar structure than in wild-type mice. Furthermore, mito-mice ND6^M^ developed honeycomb-like cysts. These histological changes were supported by Masson’s trichrome staining (Figure 2A). To quantitatively assess the severity of pulmonary fibrosis, we analyzed the Ashcroft score and hydroxyproline content in the lungs. BLM administration significantly increased the Ashcroft score and lung hydroxyproline content (Figure 2B). Both values were significantly higher in mito-mice ND6^M^ than in wild-type mice. To evaluate profibrotic protein production in the lungs, we performed Western blotting using antibodies against fibronectin, type I collagen, and α-SMA (Figure 2D). These protein expression levels were significantly higher in mito-mice ND6^M^ than in wild-type mice following BLM administration. These results show that mitochondrial dysfunction aggravates BLM-induced pulmonary fibrosis.

### 3.3. Mitochondrial Dysfunction Enhances BLM-Induced Lactate Production

To evaluate the role of mitochondria in the metabolic reprogramming induced by BLM treatment, we analyzed the production of lactate in the lungs. While BLM significantly increased lung lactate levels in both wild-type and mito-mice ND6^M^, those levels were significantly higher in mito-mice ND6^M^ than in wild-type mice (Figure 2C). This result shows BLM-induced lung lactate production is enhanced in the presence of mitochondrial dysfunction.

### 3.4. Mitochondrial Dysfunction Has a Small Effect on BLM-Induced Pulmonary Inflammation

**Figure 3:**
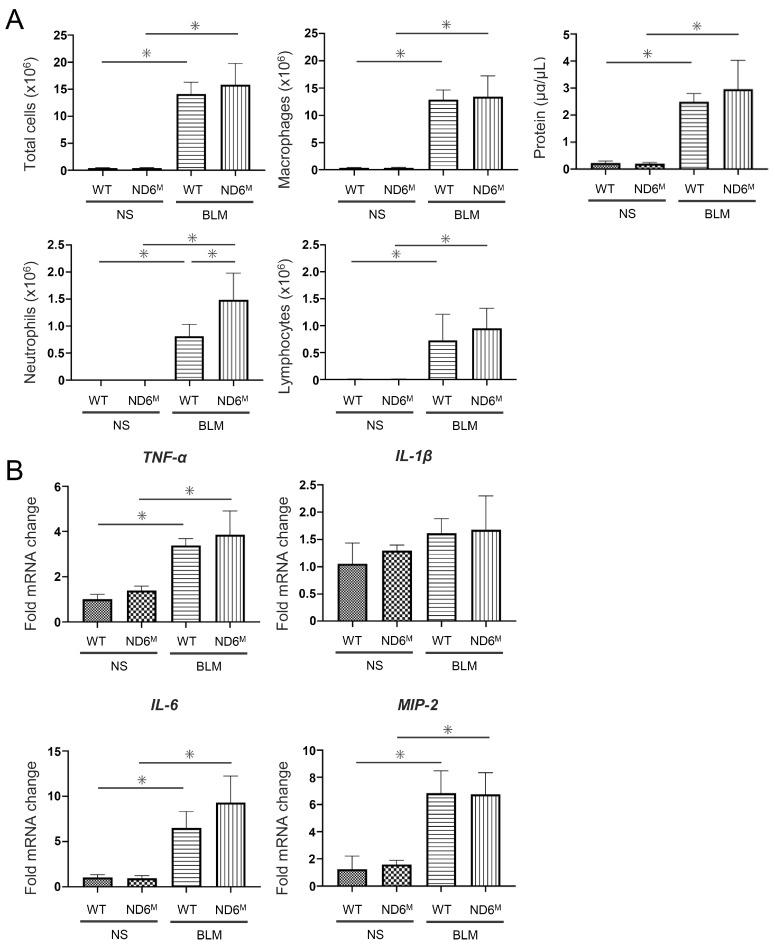
Bleomycin (BLM)-induced neutrophil increase in bronchoalveolar lavage (BAL) fluid is enhanced in mito-mice ND6^M^ without any differences in the expression of inflammatory cytokines and chemokines. Wild-type (WT) and mito-mice ND6^M^ (ND6^M^) were analyzed on day 8 after the administration of 0.1 mg/kg BLM or normal saline (NS) via oropharyngeal aspiration. (**A**) The numbers of total cells, macrophages, neutrophils, and lymphocytes, and protein levels in BAL fluids. (**B**) The mRNA expression of *TNF-α*, *IL-1β*, *IL-6*, and *macrophage inflammatory protein-2* (*MIP-2*) in the lungs. The y-axis shows the relative expression of the corresponding genes, which was normalized against *GAPDH* mRNA using the ΔΔCT method. Data are representative of two independent experiments and shown as the mean ± SD (*n* = 8). * *p* < 0.05.

To assess the influence of mitochondrial dysfunction on BLM-induced pulmonary inflammation, BAL was performed. While BLM significantly increased the number of neutrophils in both wild-type and mito-mice ND6^M^ in BAL fluid, it was significantly higher in mito-mice ND6^M^ than in wild-type mice (Figure 3A). The number of total cells, macrophages, and lymphocytes, and the protein levels in BAL fluid were not different between these mice (Figure 3A). To further examine the difference in inflammatory response between wild-type mice and mito-mice ND6^M^, mRNA expression of inflammatory cytokines and chemokines in the lungs was analyzed using real-time RT-PCR. The mRNA expression of *TNF-α*, *IL-6*, and *macrophage inflammatory protein-2* (*MIP-2*) was significantly increased following BLM inoculation and was not different between wild-type and mito-mice ND6^M^ (Figure 3B). These results suggest that mitochondrial dysfunction has a limited effect on the development of BLM-induced pulmonary inflammation.

### 3.5. Mitochondrial Dysfunction Does Not Affect M1/M2 Polarization of BMDMs

**Figure 4:**
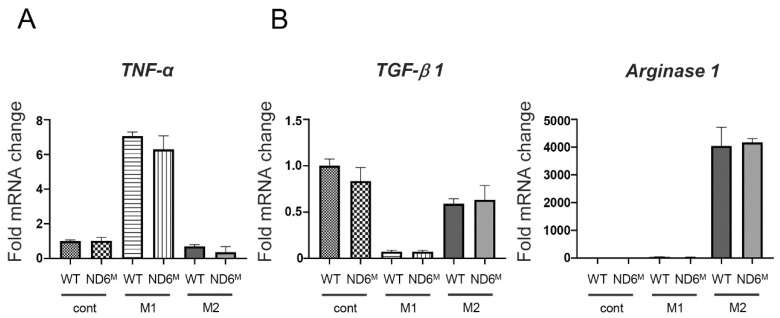
The M1/M2 polarization of bone marrow-derived macrophages (BMDMs) does not differ between wild-type and mito-mice ND6^M^. The mRNA expression of *TNF-α* (**A**), *TGF-β1*, and *arginase1* (**B**) in BMDMs generated from wild-type (WT) and mito-mice ND6^M^ (ND6^M^) and cultured in medium alone (cont) or in medium with lipopolysaccharide (LPS) plus IFN-γ (M1) (**A**) or with IL-4 (M2) (**B**) for 24 h. Data are representative of two independent experiments and shown as the mean ± SD (*n* = 6).

We examined the effect of mitochondrial dysfunction on M1/M2 polarization of BMDMs to assess the intrinsic impact of mitochondrial dysfunction on macrophage differentiation under controlled in vitro conditions. Mouse BMDMs were polarized to M1 macrophages by IFN-γ plus LPS stimulation and to M2 macrophages by IL-4 stimulation. We then analyzed the expression of genes associated with M1/M2 polarization. There was no difference in *TNF-α* mRNA expression after M1 stimulation and *TGF-β1* and *Arginase1* mRNA expression after M2 stimulation between wild-type and mito-mice ND6^M^ (Figure 4). These results suggest that mitochondrial dysfunction does not affect M1/M2 polarization of BMDMs.

### 3.6. Mitochondrial Dysfunction Promotes TGF-β1-Induced Myofibroblast Differentiation

**Figure 5:**
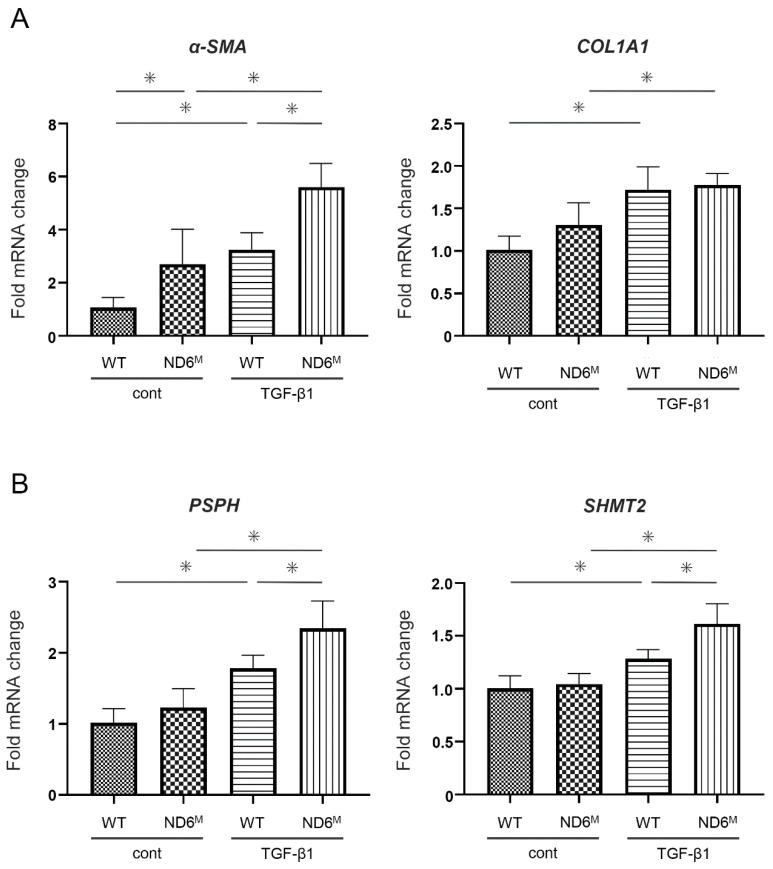
TGF-β1-induced *α-smooth muscle actin* (*α-SMA*) expression and the serine–glycine pathway activation are enhanced in lung fibroblasts from mito-mice ND6^M^. The mRNA expression of *α-SMA*, *type I collagen* (COL1A1) (**A**), *phosphoserine phosphatase* (PSPH), and *serine hydroxymethyltransferase2* (SHMT2) (**B**) in primary lung fibroblasts generated from wild-type (WT) and mito-mice ND6^M^ (ND6^M^) cultured in medium alone (cont) or in medium with TGF-β1 for 72 h. Data are representative of two independent experiments and shown as the mean ± SD (*n* = 6). * *p* < 0.05.

To examine the effects of mitochondrial dysfunction on the differentiation into myofibroblasts, primary lung fibroblasts isolated from wild-type and mito-mice ND6^M^ were treated with TGF-β1. TGF-β1 significantly upregulated mRNA expression of *α-SMA and type I collagen* (*COL1A1*). While α-SMA expression was significantly higher in mito-mice ND6^M^ than in wild-type mice, COL1A1 expression was not different between these mice (Figure 5A). These results suggest that mitochondrial dysfunction promotes *α-SMA* expression induced by TGF-β1.

Next, to investigate the effect of mitochondrial dysfunction on the metabolic process involved in myofibroblast differentiation, we focused on the serine–glycine pathway, which begins with 3-phosphoglycerate, an intermediate product of glycolysis, and contributes to collagen synthesis. The mRNA expression of *phosphoserine phosphatase* (*PSPH*) and *serine hydroxymethyltransferase2* (*SHMT2*), two of the serine–glycine metabolic enzymes involved in this pathway (Figure 5B), was induced by TGF-β1 and was significantly higher in mito-mice ND6^M^ than in wild-type mice. Taken together, these results suggest that mitochondrial dysfunction promotes TGF-β1-induced myofibroblast differentiation by enhancing *α-SMA* expression and by activating the serine–glycine pathway.

## 4. Discussion

In cells from IPF patients, the following features of mitochondrial dysfunction have been identified alongside other senescence-associated phenotypes: reduced activity of the respiratory chain complex, impaired mitochondrial biogenesis, decreased mitochondrial quality control, mtDNA damage, and increased mitochondrial ROS production [23]. Thus far, studies investigating the direct impact of mitochondrial dysfunction on pulmonary fibrosis are limited. In mice deficient in PTEN-induced kinase 1 or sirtuin-3, both of which are necessary for maintaining mitochondrial homeostasis, pulmonary fibrosis worsened with the impairment of mitochondrial function [7,24,25]. Han et al. demonstrated that alveolar epithelial cells lacking Ndufs2, a complex I subunit encoded by nuclear DNA, accumulate transitional epithelial cell populations exhibiting characteristics of both type I and type II alveolar epithelial cells (AECs), thereby impairing normal alveolar epithelial differentiation and repair [26]. Furthermore, such transitional epithelial cells have been reported in patients with pulmonary fibrosis in recent years [27]. By employing mito-mice ND6^M^ in the BLM-induced pulmonary fibrosis model, our study has clarified the effect of mitochondrial dysfunction alone on the development of pulmonary fibrosis. To our knowledge, this is the first report demonstrating that mitochondrial dysfunction promotes BLM-induced pulmonary fibrosis and myofibroblast differentiation. The impact of mitochondrial dysfunction on mortality clearly plays a crucial role for mitochondria in this fibrosis model.

In IPF patients and pulmonary fibrosis models, increased glycolysis and glucose uptake, as well as reduced OXPHOS, have been reported in myofibroblasts [28,29,30,31]. In BLM-induced pulmonary fibrosis models, hypoxia-inducible factor 1 (HIF1-α) exacerbates pulmonary fibrosis and myofibroblast differentiation through the activation of glycolysis [28,32]. These findings suggest a critical role for glycolysis in myofibroblast differentiation. TGF-β1 induces myofibroblast differentiation by enhancing glycolysis and mitochondrial biogenesis [33]. Inhibiting glycolysis with 2-deoxy-D-glucose or suppressing mitochondrial biogenesis by silencing mitochondrial transcription factors reduces TGF-β1-induced α-SMA expression without affecting COL1A1 or fibronectin expression, suggesting that α-SMA expression in fibroblasts may be associated with cellular bioenergetic status [33]. Enhanced glucose uptake and the activation of the serine–glycine pathway were suggested to supply glycine, a collagen synthesis precursor, in TGF-β1-treated pulmonary fibroblasts [34]. In our study, mitochondrial dysfunction activates glycolysis, as suggested by increased lactate production, and enhanced TGF-β1-induced α-SMA expression and the serine–glycine pathway activation without changing COL1A1 expression. These results suggest that mitochondrial dysfunction promotes TGF-β1-induced α-SMA expression through the activation of glycolysis. Notably, glycine synthesis has been reported to promote collagen production via post-transcriptional mechanisms [35,36]. Increased glycine synthesis activity may therefore enhance collagen protein levels without necessarily changing collagen mRNA expression. This concept is consistent with our observation that type I collagen protein levels in the lungs were elevated, whereas COL1A1 mRNA expression in primary lung fibroblasts remained unchanged in the presence of mitochondrial dysfunction. While mitochondrial dysfunction does not directly enhance TGF-β1-induced ECM expression, it may contribute to increasing ECM production by providing glycine. Further studies are required to clarify the molecular mechanisms by which metabolic reprogramming regulates the process of myofibroblast differentiation.

The respiratory chain activity, including complex I, is reduced in IPF patients [23,37]. While there are few reports on the role of subunits of respiratory chain complex I in IPF, ND6 is known to play a crucial role in the assembly and structural stability of the complex I membrane arm, unlike other complex I subunits [38]. Impairment of ND6 destabilizes the enzyme, disrupting mitochondrial respiratory function [39]. In our study, in line with previous reports [16,18], lung lactate production was not increased in ND6^M^ at baseline, suggesting ND6^M^ maintains metabolic compensation at rest. Under BLM-induced stress conditions, ND6^M^ enhances glycolytic activation and lactate production, which has been reported to play a causative role in the progression of fibrosis [40]. In IPF lungs, increased glycolysis leads to elevated lactate production and lactate dehydrogenase 5 (LDH5) expression [8]. The local pH decrease due to lactate production activates TGF-β, which further induces the expression of HIF1-α and LDH5, and their synergistic effects promote the differentiation into myofibroblasts [8,40]. Although the source of lactate in the lungs of pulmonary fibrosis remains unclear, AECs and fibroblasts have been suggested as its primary source [8,41]. Moreover, in mito-mice ND6^M^, increased ROS may further activate TGF-β1 [42]. Therefore, multiple metabolites resulting from mitochondrial dysfunction may collectively activate TGF-β1 and thereby promote BLM-induced pulmonary fibrosis in mito-mice ND6^M^.

Alveolar macrophages are reported to polarize into the M2 phenotype and contribute to the progression of IPF by producing TGF-β and CCL18 [3]. Studies using BMDMs have reported that glycolysis is necessary for the polarization toward M1 macrophages, while fatty acid oxidation (FAO) and OXPHOS are required for the polarization toward M2 macrophages [9,43]. However, recent studies have shown that nitric oxide (NO), produced by macrophages, is involved in M1 polarization independently of OXPHOS activity [44] and inhibits the repolarization from M1 to M2 [45]. Additionally, several studies have suggested that glycolysis is necessary for maintaining M2 polarization [46,47]. In this study, mitochondrial dysfunction had no effect on the polarization of BMDMs, suggesting that macrophage polarization may not be solely determined by mitochondria-dependent metabolic reprogramming. Although reports on mitochondrial function in lung diseases are limited, OXPHOS is reduced in macrophages in BAL fluid from IPF patients [48]. Furthermore, M2-like polarization, observed in alveolar macrophages in mice with pulmonary fibrosis, was dependent on glycolysis rather than FAO or OXPHOS [49]. These findings include points that do not align with classical M1/M2 macrophage differentiation. Further research is required to clarify how cellular metabolism regulates the polarization of alveolar macrophages in the development of pulmonary fibrosis.

While the prognosis of IPF has been shown to correlate with neutrophil counts in BAL fluid and peripheral blood [50,51], the role of neutrophils in pulmonary fibrosis remains unclear. Failure to effectively recruit neutrophils into the lungs in formyl peptide receptor 1-deficient mice attenuates BLM-induced pulmonary fibrosis, suggesting a potential contribution of neutrophils to the development of fibrosis [52]. In this study, mitochondrial dysfunction increased BLM-induced neutrophil recruitment in BAL fluid without changing the expression of neutrophil-associated cytokines or chemokines. Recent studies have demonstrated that neutrophil extracellular traps (NETs), fibrous structures released from activated neutrophils, and S100A8/A9, a calcium-binding protein mainly produced by neutrophils, enhance fibroblast proliferation and myofibroblast differentiation [53,54]. These results suggest that neutrophils may promote fibrosis through their interaction with fibroblasts. Further studies are required to clarify the mechanisms and roles of increased neutrophil recruitment in mito-mice ND6^M^ following BLM inoculation.

This study has several limitations. First, this study focused on myofibroblast differentiation and macrophage polarization, leaving out the evaluation of alveolar macrophages, neutrophils, and AECs. To analyze the effects of mitochondrial dysfunction on these cells, cell-specific analyses, such as single-cell RNA-seq, are required. In particular, given recent reports that mitochondrial dysfunction in alveolar epithelial cells induces transitional epithelial cell states, it remains possible that similar transitional epithelial populations may also be present in mito-mice ND6^M^, although this was not examined in the present study. Fibrosis-related cytokines and chemokines in BAL fluid were also not measured, which might provide complementary information on the profibrotic microenvironment. Second, the effect of improving mitochondrial function on pulmonary fibrosis has not been confirmed. Further studies using reagents that improve mitochondrial function are required to investigate their therapeutic potential against this disease.

In conclusion, this study demonstrates that mitochondrial dysfunction exacerbates pulmonary fibrosis and increases mortality. Mitochondrial dysfunction may promote pulmonary fibrosis by enhancing the differentiation of fibroblasts into myofibroblasts. Further research into the role of mitochondria in pulmonary fibrosis may improve our understanding of this disease and lead to the development of novel therapeutic strategies.

## Figures and Tables

**Table 1:** Sequences of primers used for real-time reverse transcription PCR.

Target	Sequences
GAPDH	5′-CCGCATCTTCTTGTGCAGTG-3′ (forward)
	5′-CGTTGATGGCAACAATCTCC-3′ (reverse)
TNF-α	5′-CCCTCACACTCAGATCATCTTCT-3′ (forward)
	5′-GCTACGACGTGGGCTACAG-3′ (reverse)
IL-6	5′-TAGTCCTTCCTACCCCAATTTCC-3′ (forward)
	5′-TTGGTCCTTAGCCACTCCTTC-3′ (reverse)
MIP-2	5′-CCAACCACCAGGCTACAGG-3′ (forward)
	5′-GCGTCACACTCAAGCTCTG-3′ (reverse)
TGF-β1	5′-CTCCCGTGGCTTCTAGTGC-3′ (forward)
	5′-CGCAGCTCTAGGAGCATGTG-3′ (reverse)
Arginase1	5′-CTCCAAGCCAAAGTCCTTAGAG-3′ (forward)
	5′-AGGAGCTGTCATTAGGGACATC-3′ (reverse)
COL1A1	5′-GCTCCTCTTAGGGGCCACT-3′ (forward)
	5′-CCACGTCTCACCATTGGGG-3′ (reverse)
α-SMA	5′-GTCCCAGACATCAGGGAGTAA-3′ (forward)
	5′-TCGGATACTTCAGCGTCAGGA-3′ (reverse)
PSPH	5′-AGGAAGCTCTTCTGTTCAGCG-3′ (forward)
	5′-GAGCCTCTGGACTTGATCCC-3′ (reverse)
SHMT2	5′-TGGCAAGAGATACTACGGAGG-3′ (forward)
	5′-GCAGGTCCAACCCCATGAT-3′ (reverse)

Definitions of abbreviations: MIP-2, macrophage inflammatory protein 2; COL1A1, type I collagen α1; α-SMA, α-smooth muscle actin; PSPH, phosphoserine phosphatase; SHMT2, serine hydroxymethyltransferase2.

## Data Availability

The original contributions presented in this study are included in the article/Supplementary Material. Further inquiries can be directed to the corresponding author.

## Supplementary Materials

The following supporting information can be downloaded at: https://www.mdpi.com/article/10.3390/cells15070582/s1, Table S1: Results of normality testing for hydroxyproline and lactate in the lungs; Table S2: Results of normality testing for the mRNA expression in primary lung fibroblasts.

## Abbreviations

The following abbreviations are used in this manuscript:

IPF, idiopathic pulmonary fibrosis; ATS, American Thoracic Society; ERS, European Respiratory Society; JRS, Japanese Respiratory Society; ALAT, Latin American Thoracic Association; α-SMA, α-smooth muscle actin; ECM, extracellular matrix; OXPHOS, oxidative phosphorylation; ROS, reactive oxygen species; MtDNA, mitochondrial DNA; BLM, bleomycin; NS, normal saline; BAL, bronchoalveolar lavage; BMDMs, bone marrow-derived macrophages; DMEM, Dulbecco’s modified Eagle’s medium; FBS, fetal bovine serum; LPS, lipopolysaccharide; real-time RT-PCR, real-time reverse transcription PCR; MIP-2, macrophage inflammatory protein 2; COL1A1, type I collagen; PSPH, phosphoserine phosphatase; SHMT2, serine hydroxymethyltransferase2; AECs, alveolar epithelial cells; HIF1-α, hypoxia-inducible factor 1; LDH5, lactate dehydrogenase 5; FAO, fatty acid oxidation; NO, nitric oxide; NETs, neutrophil extracellular traps.
