# Supplementary material for: Mitochondria-Dependent Metabolic Reprogramming Enhances Myofibroblast Differentiation and Aggravates Bleomycin-Induced Pulmonary Fibrosis
Source: Cells. 2026 Mar 25;15(7):582. doi: 10.3390/cells15070582 (PMC13072105; doi:10.3390/cells15070582)
Supplement: Supplementary file 1 [file cells-15-00582-s001.zip › Supplement; Table S1-S2 revised 010626.pdf]

Table S1. Results of normality testing for hydroxyproline and lactate in the lungs

Variables	Statics	*p* value
Hydroxyproline		
WT + NS	0.966	0.848
$ND6^M$ + NS	0.931	0.601
WT + BLM	0.936	0.601
$ND6^M$ + BLM	0.938	0.596
Lactate		
WT + NS	0.957	0.600
$ND6^M$ + NS	0.958	0.607
WT + BLM	0.989	0.995
$ND6^M$ + BLM	0.925	0.471

Definitions of abbreviations: WT, Wild-type; NS, Normal saline; $ND6^M$, mito-mice $ND6^M$; BLM, Bleomycin. The variables were considered normally distributed with p-values ≧ 0.05 in the Shapiro–Wilk test. Bold indicates significance (p<0.05).

Table S2. Results of normality testing for the mRNA expression in primary lung fibroblasts

Variables	Statics	*p* value
α-SMA		
WT	0.961	0.824
ND6^{M}	0.981	0.957
WT + TGF-β1	0.897	0.357
ND6^{M} + TGF-β1	0.886	0.104
COL1A1		
WT	0.806	0.067
ND6^{M}	0.962	0.831
WT + TGF-β1	0.967	0.870
ND6M + TGF-β1	0.940	0.661
PSPH		
WT	0.928	0.561
ND6^{M}	0.944	0.691
WT + TGF-β1	0.913	0.457
ND6^{M} + TGF-β1	0.934	0.626
SHMT2		
WT	0.907	0.418
ND6^{M}	0.988	0.983
WT + TGF-β1	0.900	0.373
ND6^{M} + TGF-β1	0.895	0.383

Definitions of abbreviations: α-SMA, α-smooth muscle actin; WT, Wild-type; ND6^{M}, mito-mice ND6^{M}; COL1A1, Type I collagen; PSPH, Phosphoserine phosphatase; SHMT2, Serine hydroxymethyltransferase2. The variables were considered normally distributed with p-values ≧ 0.05 in the Shapiro–Wilk test. Bold indicates significance (p<0.05).
